# Long term cardiometabolic outcomes in COVID positive patients

**DOI:** 10.1177/22799036261445801

**Published:** 2026-06-10

**Authors:** Lauren Chetty, Poovendhree Reddy, Sapna Ramdin, Nalini Govender

**Affiliations:** 1 Department of Community Health Studies, Faculty of Health Sciences, 56391 Durban University of Technology, Durban, South Africa; 2 Doctoral Research Office, 128659 Mancosa, Durban, South Africa; 3 Department of Basic Medical Sciences, Faculty of Health Sciences, 56391 Durban University of Technology, Durban, South Africa

**Keywords:** long COVID, post COVID-19, new onset diabetes mellitus, new onset hypertension, cardiometabolic effects, long COVID effects

## Abstract

In South Africa, approximately 4.5 million confirmed COVID-19 cases and 103,000 deaths have been noted. Symptoms range from being asymptomatic, mild respiratory issues to severe multi-organ failure and consequent death. Cardiometabolic risk factors, viz., type 1 diabetes mellitus (T1DM) and type 2 diabetes mellitus (T2DM), atherosclerosis, chronic kidney disease, hypertension, heart failure, and obesity, are flagged as the most prevalent comorbidities associated with the risk of severe COVID-19 and death. Many who have recovered from hospitalization continue to experience lingering symptoms known as long COVID, which have been linked to new-onset cardiometabolic disorders (CMD). This narrative review thus focusses on the clinical factors and the patient burden of diabetes mellitus and hypertension as two of the most commonly observed CMD, and aims to raise awareness regarding possible cardiometabolic complications. The findings expand the existing literature on the cardiometabolic effects of COVID-19, concentrating on outcomes observed more than three months after the acute phase of the illness.

## 1. Introduction

Globally, approximately 6.92 million fatalities associated with SARS-CoV-2 infections have been reported, of which 103,000 reside within South Africa.^1^ A large proportion of SARS-CoV-2 infected individuals experienced asymptomatic or mild respiratory conditions extending to severe multi-organ dysfunction and mortality.^2,3^ However, a substantial number of affected patients continue to experience symptoms associated with the COVID-19 infection, referred to as long COVID.^4,5^ The Centers for Disease Control and Prevention (CDC) defines “long COVID” by symptoms that persist for at least four weeks beyond the initial acute phase of infection.^6^ In contrast, long COVID was redefined by the World Health Organisation (WHO), as new or ongoing symptoms that manifest three months after the initial infection.^7^ According to the National Institutes of Health (NIH), symptoms linger for weeks, months, or even years following the initial COVID-19 diagnosis.^8^

The definition of long COVID varies between studies, however, several investigators define long COVID as the emergence of new related symptoms and signs following the initial infection with SARS-CoV-2 or the persistence of symptoms over four weeks, relapse, and/or progress.^9,10^ Likewise, others suggest that the symptoms of this disease can persist for three to twelve weeks, while chronic COVID-19 symptoms manifest beyond twelve weeks.^10–12^ Cardiometobolic disorders are the most prevalent and are defined as a collection of interrelated symptoms that result in diabetes, cardiovascular disease (CVD), and chronic renal failure, with a 40% odds for diabetes and 200% for end-stage kidney disease.^13,14^ Of note, diabetes mellitus and hypertension are amongst the most common cardiometabolic diseases linked to long COVID.^15–18^ The findings based on a population of 906,849 hospitalized COVID-19 patients indicate that 25.5% of this population were hypertensive (95% UI, 23.6–27.4), followed by 20.5% being diagnosed with diabetes mellitus (95% UI, 18.9–22.1).^15^ Findings from a meta-analysis of 20 cohort studies comprising 60,221,176 patients,^16^ revealed a statistically significant association between long COVID and the risk of diabetes, with those affected demonstrating a 46% increased risk (HR = 1.45; 95% CI: 1.38–1.55, P < 0.001).^16^ Likewise, data from a systematic review and meta-analysis of 19,293,346 patients, revealed a strong association between hypertension (HR=1.70; 95% CI: 1.46-1.97) and long COVID, which was greater than that reported for heart failure (HR=1.40).^18^ As we approach the sixth year since the emergence of SARS-CoV-2, the understanding of its pathophysiology continues to evolve. Given the large number of people who have recovered from COVID-19, the long-term effects of acute COVID-19 recovery pose a significant health concern and could have significant financial and medical implications.^9,10^ However, the long-term impact (>1 year) of COVID-19 on an individual’s health remains unclear.^19–21^ Noteworthy, literature refers to the clinical impact as long COVID, long-haul COVID-19, post-acute COVID-19, long-term effects of COVID-19, chronic COVID-19, and post-acute sequelae of SARS-CoV-2.^22^ Despite the use of several terms, it remains as a single clinical entity and warrants the prioritization of accurate and comprehensive data collection.^22^

Globally the COVID-19 pandemic did pose a significant challenge to healthcare systems, as the affected individuals require continuous medical support for prolonged periods.^23^ The emergence of long COVID has subsequently challenged the healthcare infrastructure, which now requires substantial investments in infrastructure, increased financial support, and robust international collaboration are paramount.^24^ In Africa, these challenges include limited healthcare infrastructure, a shortage of medical professionals, and inadequate access to healthcare services, especially in rural regions.^25^ The economic impact is significant particularly in the informal sector, which accounts for a large proportion of the workforce.^26^ This combined with the high prevalence of HIV, tuberculosis, and other related comorbidities, exacerbates the long COVID symptoms, and requires the integration of COVID-19 care with existing healthcare services.^27^ Social (i.e., stigma and discrimination) and cultural factors can significantly impact healthcare-seeking behaviors and access to essential services.^28^ The far-reaching social and economic consequences of long COVID, such as loss of income, social isolation, and stigma, necessitate the provision of comprehensive support services such as mental health care and social support.^29^ In light of the various consequences reported thus far, this narrative review evaluated the clinical factors and patient burdens associated with the emergence of new non-communicable diseases post the COVID-19 pandemic.

## 2. Methodology

An online search of all published studies between 1 March 2020 and 31 December 2024, was done using electronic scientific search engines viz., PubMed, Google Scholar, Web of Science, Elsevier, and Science Direct. The MeSH terms used for identifying the relevant studies included “non-communicable diseases”, “new onset, diabetes”, “ new onset hypertension”, “health-related quality of life”, “incidence”, “post COVID”, “COVID-19”, and “long COVID”. Articles of interest was filtered based on the titles and abstracts. Only articles that were directly related to cardiometabolic outcomes in COVID positive patients and published in English, was reviewed. This included meta-analysis, observational studies, systematic reviews, case reports and longitudinal studies. Relevant articles of interest were also identified by screening reference lists of all included articles.

## 3. Epidemiology and pathophysiology of long COVID

### 3.1. Epidemiology

The incidence and mortality rates of COVID-19 vary between countries, which makes it difficult to accurately predict the number of patients who will develop long COVID.^30^ Furthermore, the precise reporting of long COVID presents challenges which may be attributed to variations in the underlying population, diagnostic accuracy, reporting mechanisms, and the capabilities of healthcare systems.^30^ While the precise epidemiological data on long COVID remains elusive, its acquisition is crucial in guiding healthcare systems and governments in the formulation of effective support and treatment protocols.^30^ The volume of published literature describing cases of patients with COVID-19 who subsequently develop long COVID symptoms is continually growing,^30–32^ which will allow for an improved understanding of its epidemiology.^30^ Variations in follow-up examination times post COVID-19 infection have been reported globally.^33,34^ In Michigan, an observational cohort study involving 1648 patients with COVID-19 admitted to 38 hospitals, revealed that within 60 days of discharge, 189 (15.1% of hospital survivors) were hospitalized.^33^ Likewise, an online survey obtained 3762 participants with confirmed or suspected COVID-19 from 56 countries found that almost 91% experienced a recovery time exceeding 35 weeks and approximately 65 million cases were affected by long COVID.^34^

Noteworthy, SARS-CoV-2 impacts an estimated 35% of outpatients, and approximately 87% of hospitalized individuals, with a greater risk of infection amongst advanced age individuals and those with underlying comorbidities.^12^ Comorbidities that increase the vulnerability include cardiometabolic disorders such as type 2 diabetes mellitus (T2DM), hypertension, and dyslipidemia.^35^ This was corroborated by Barbu and coworkers, who reported the incidence of hypertension (43.1%), diabetes (33.2%), and/or coronary heart disease (26.0%) 3 to 6 months post COVID-19 infection.^36^ Moreover, data from a six-year Italian study comprising of 228,266 individuals, revealed a 29% (OR=1.29; 95% CI, 1.19–1.39) increased risk of the development of dyslipidemia during 2020-2022 of the pandemic compared to the pre-pandemic periods (2017-2019).^37^ This data demonstrates a significant increase in dyslipidemia cases during the pandemic, emphasising the importance of clinical monitoring protocols among COVID-19 survivors to prevent dyslipidemia.^37^ Furthermore, the prevalence of dyslipidemia in Spanish patients with T2DM and/or dyslipidemia rose from 23.5% (n=310,796) in 2019 to 23.6% (n=314,155) in 2020.^38^ This observational retrospective study also documented an additional increase of 24.5% (n=324,639) in 2021 (p<0.001).^38^ The incidence of dyslipidemia increased from 11.03 per 100,000 individuals in 2020 to 18.3 per 100,000 individuals in 2021 (p<0.001).^38^ Interestingly, Liang and coworkers stated that COVID-19 is not directly associated to dyslipidemia.^39^ Although some research has suggested a relationship between COVID-19 and an increased prevalence of dyslipidemia, it is likely that this relationship is influenced by the pre-existing dyslipidemic conditions caused by COVID-19.^39^ Globally, dyslipidemia is reported as a common risk factor for atherosclerotic cardiovascular disease and is often associated with sedentary lifestyles, smoking, high-calorie diets, diabetes mellitus, and obesity, risk patterns that manifested during the COVID-19 pandemic.^40^ Despite the data availability regarding the long-term effects of COVID-19 on pre-existing non communicable diseases (NCD),^41–44^ limited epidemiological data on the incidence of NCDs post COVID-19 infection is documented.^45,46^ The scarcity of comprehensive data regarding the potential risks and clinical impact of diabetes and hypertension, within the long COVID phase is concerning and may affect how effective management can be provided.^43,47–49^ Notably, Swarnakar and Yadav corroborated that the lack of long-term clinical and scientific data on the healthcare system or individuals with long COVID is disastrous.^50^ It is posited that having access to such data will expedite the development of public health policies and programs for effective risk assessment, surveillance, patient follow-up, and prevention.^51,52^ This will subsequently enable a more inclusive approach to safeguarding the population from the dual burden of communicable and non-communicable diseases.^51^

### 3.2. Pathophysiology

**Figure 1.**
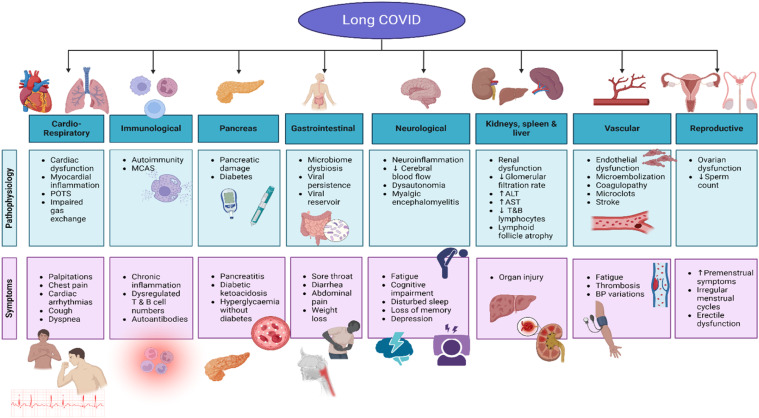
Pathophysiological mechanisms and symptoms associated to “long COVID” (Adapted from Raveendran et al., 2021).^12^ Multi-organ injury characterised by dysregulation of the respiratory, cardiovascular, neurological, digestive, circulatory, musculoskeletal, and reproductive systems contribute to the symptoms associated with long COVID. This figure was generated using BioRender (https://biorender.com/).

The precise pathophysiological mechanisms of this disease remain undefined.^53,54^ The acute phase of COVID-19 compromises organ function, exacerbating the vulnerability to developing long COVID, and persistent multiorgan symptoms.^55,56^ Multiple factors are involved in the development of long COVID symptoms as shown in Figure 1.^12^ Immune dysregulation is a key underlying mechanism, leading to viral persistence, microbiota disruption, autoimmunity, endothelitis, metabolic dysregulation, and post-intensive care syndrome.^12,56,57^ Immune profiles indicative of persistent inflammation, vascular injury, and immune cell differentiation were observed in affected individuals two to eight months post-infection.^56^ Higher cytokine concentrations viz. interleukin 6 (IL-6), suggestive of sustained multiorgan injury were observed in these patients compared to healthy individuals.^56^ Physiologically, IL-6 crosses the blood-brain barrier and may modify neuronal activity thereby inducing neural complications such as dysautonomia, depression, and hearing loss.^25^ Patients display lymphopenia, lymphocyte activation and dysfunction, granulocyte and monocyte abnormalities, elevated cytokine levels, as well as an increase in immunoglobulin G (IgG) and total antibodies,^58^ these aspects are immune characteristics that are potential therapeutic targets for COVID-19. In lymphopenia, particularly in severe cases, increased expressions of CD69, CD38, and CD44 are noted on CD4^+^ and CD8^+^ T cells, and virus-specific T cells, all of which indicate a central memory phenotype with elevated IFN-γ, TNF-α, and IL-2 levels.^58^ Lymphocytes also demonstrate a phenotype with increased expression of programmed cell death protein-1 (PD1), T cell immunoglobulin domain and mucin domain-3 (TIM3), and killer cell lectin-like receptor subfamily C member 1 (NKG2A).^58^ Neutrophil and IL-1β, IL-6, and IL-10 cytokine levels are elevated in severe cases, with a reduction in eosinophils, basophils, and monocytes. Additionally, there is notable elevation in IgG levels and total antibody titers,^58^ indicative of an integral role of immune dysregulation in the development of long COVID. Multiple mechanisms have been proposed to explain long COVID which demonstrate similarities with post-viral syndromes and conditions like ME/CFS.^59^ The main hypotheses however include incomplete viral clearance, latent virus reactivation, immune dysregulation and autoimmunity, microbiome imbalances, endothelial dysfunction and coagulation problems and mitochondrial dysfunction.^53,59^ Persistent symptoms however, may stem from immune dysregulation.^59^ An earlier report highlighted an association between long COVID and lower levels of cytotoxic T lymphocytes (CTLs) and natural killer (NK) cells, suggestive of viral persistence and increased disease severity.^60^ Furthermore, impaired oxidative phosphorylation (OXPHOS) in peripheral blood mononuclear cells are associated to fatigue and cognitive symptoms.^61^ Systemic inflammation on a regular basis may result in structural cardiac abnormalities, fibrosis, and a higher risk of heart failure or arrhythmia.^62^ Moreover, autoimmune disorders that involve antiphospholipid antibodies, may increase vascular inflammation and risk of blood clots.^63^ In addition, the SARS-CoV-2 can also damage the kidneys by affecting ACE-2 receptor function, resulting in renal insufficiencies, especially in patients with risk factors.^10,64,65^

## 4. Clinical manifestations of long COVID: Potential mechanisms

Findings from a global online study evaluating responses from 3762 participants with confirmed or suspected COVID-19, and with the disease lasting over 28 days and onset prior to June 2020, documented an average of 55.9±25.5 symptoms experienced.^34^ Notably, about 203 symptoms associated with 10 organ systems were reported, with 66 symptoms traced over seven months. Fatigue, post-exertional malaise, and cognitive dysfunction were more frequently reported after month 6, however the prevalence fluctuated over time.^34^ In 2021, participants from a prospective long COVID cohort study (n=201) reported reduced functionality of the pancreas (40%), liver (28%), heart (26%), lung (11%), kidney (4%), and spleen (4%).^66^ Notably, 29% of patients exhibited multiorgan impairment after a median of 141 days post long COVID infection.^66^ Cases of severe long COVID are characterised by an increase in liver volume, independent of obesity, hypertension, diabetes mellitus, and cardiovascular conditions, indicative of myocarditis, pancreatitis and fat accumulation.^66^ Other systemic anomalies include, thrombotic and cerebrovascular diseases.^67–69^

The susceptibility of endocrine system is associated with the vascular supply and increased expression of the angiotensin-converting enzyme 2 (ACE2) receptor and TMPRSS2 proteins responsible for the cellular entry of the SARS-CoV-2 virus.^70^ Most cases reported thus far are associated with active COVID-19 infection, with only some cases following recovery from COVID-19.^70^ Regardless of whether its during the active or post infection phase, headaches and visual disturbances are commonly reported together with anterior pituitary hormone deficiencies.^70^ Some patients were managed by emergent transsphenoidal surgery, and have experienced successful outcomes, however, postoperative follow-up data is unavailable.^71^ Furthermore, COVID-19 related inflammation in the pituitary gland results in hormone imbalances and consequent alterations in blood glucose and blood pressure regulation.^72^ Cushing disease can also increase the risk of diabetes and hypertension post COVID-19.^73^ Moreover, COVID-19 disrupts glycemic control in individuals with diabetes, raising the incidence and severity of new onset diabetic ketoacidosis.^73^ The viral entry into the pancreatic beta cells via ACE2 receptors, interrupts insulin secretion resulting in new onset diabetes mellitus.^73^ Moreover, adrenal insufficiency secondary to acute adrenal infarction^74^ and adrenal hemorrhage^75,76^ have been described in case reports following COVID-19. However, underlying comorbidities, such as antiphospholipid syndrome, may have been contributory in some individuals. Albeit adrenal function remains normal in most patients post COVID-19,^69^ fatigue, postural hypotension, and cognitive impairment are frequently reported by patients with long COVID and those with adrenal insufficiency.^77^ Whilst symptoms of long COVID have similarity to those of adrenal insufficiency, there remains little robust evidence of glucocorticoid deficiency, even in patients treated with dexamethasone.^69^ Elevations in cortisol levels in approximately 70% of COVID-19 patients may have causautive roles in adrenal gland dysfunction in long COVID.^78^ Similar rates (60%) were observed in COVID-19 patients developing insulin resistances, thus increasing the risk of T2DM.^79^

In another study, abnormal thyroid function was noted in 64% of patients (n=32) diagnosed with COVID-19 over a follow-up period of 3 months.^80^ Among these individuals, 56% demonstrated lower thyroid stimulating hormone (TSH) and triiodothyronine (T3) levels, when compared to a healthy control group, albeit no significant variations in thyroxine (T4) levels.^80^ Cardiometabolic consequences occur in 30 to 50% of COVID-19 patients with thyroid gland dysfunction.^81^ Findings from a prospective cohort study involving hospitalized, male COVID-19 patients (n= 221, aged >18 years), revealed that 113 (51.1%) had hypogonadism (serum total testosterone level <300 ng/dL).^82^ Moreover, an observational study involving 43 sexually active male patients who had recovered from COVID-19, revealed that 25.6% exhibited oligo-crypto-azoospermia.^83^ Limited data exist regarding the impact of COVID-19 infection on ovarian function, aside from the non-infectious consequences viz., heightened psychological stress and weight gain.^83^ The lack of data emphasises the need to enhance the current understanding of how endocrine dysfunction leads to long COVID.^83^

### 4.1. Cardiometabolic outcomes associated with COVID-19

Cardiometabolic risk factors, including T1DM and T2DM, atherosclerotic cardiovascular disease, chronic kidney disease, hypertension, heart failure and obesity are common comorbidities associated with the risk of long COVID and mortality.^84^ More than 40% of adults in the United States reported having COVID-19 in the past, and one in five continue to experience symptoms.^85,86^ During the acute phase of COVID-19, and following an acute COVID-19 infection, an increased incidence of CMD ranging from T2DM to CVD such as hypertension, has been reported in observational studies.^87,88^ Associations between new-onset CMD post COVID-19 conditions continue to emerge.^89,90^ Understanding the magnitude of outcomes and distinguishing between incident conditions such as diabetes, hypertension, and dyslipidemia, and pre-existing comorbidities are crucial for risk stratification and health system planning.^91^ Findings from a meta-analysis of 20 cohorts comprising 60 million individuals revealed a significant association between COVID-19 infection and increased risk of new-onset diabetes with a Hazard Ratio (HR) of 1.46, (95% CI 1.38-1.55), and persisting for up to 2 years.^16^ Furthermore, post hospitalisation, recovering COVID-19 patients present with notable increases in LDL cholesterol and triglyceride levels 3-6 months post-discharge.^92^ Similarly, the risk of new-onset hypertension is increased in the post-acute COVID-19 phase (6-8 months; HR=1.70; 95% CI: 1.46-1.97).^17^ These findings indicate that such incident conditions dominate the public health issues associated with long COVID, whereas pre-existing comorbidities amplify severity and delay recovery.^17^ Bowe et al., (2021) investigated the risks of complications in a cohort study of 1,726,683 US Veterans, inclusive of 89,216 COVID-19 survivors.^93^ The data indicate an increased risk of acute kidney infection (HR=1.94, 95% CI: 1.86-2.04) beyond the acute illness phase, and the severity correlated with the risk of adverse kidney outcomes.^93^ A more recent Italian study in alignment with the American Heart Association, introduced a term called cardiovascular-kidney-metabolic syndrome (CKM), which describes the complex interplay among health conditions linking heart, kidney, and metabolism, demonstrates a significant surge in CKM conditions during the COVID-19 pandemic.^94^ This study evaluated a primary care database consisting of a cohort of 81051 participants over 6 years, 32650 in the pre-pandemic phase (2017-2019), and 48401 during the pandemic period (2020-2022).^94^ Findings from this study confirms an increase in adults having ≥1 CKM condition, which rose from 34% to 44%, and driven by increases in T2D from 3.6% to 12%, and comorbid CVD T2D from 1.3% to 3.4%, and CKD T2D from 3.4% to 5.0%.^94^ Notably, the rates increased from 10.8% to 13.8% among adults with ≥2 CKM conditions. These findings emphasize the need for optimized care pathways in response to the impact caused by the pandemic on CKM syndrome prevalence, targeting management of T2D mainly.^94^ A meta-analysis of 21,463,173 patients, including 1,628,424 with confirmed COVID-19 infection, highlights a pooled incidence of 1.1% for long COVID heart failure (HF) (95% CI: 0.7-1.6).^95^ The data suggests a 90% increased risk of incident HF (HR=1.90, 95% CI: 1.54-3.24) in COVID-19 survivors compared to non-infected controls.^95^

Noteworthy, the absolute burden of cardiometabolic conditions following COVID-19 infection is estimated to range from 1% to 4%.^96^ Given the large number of people affected by COVID-19, this translates to millions of individuals worldwide with potential long-term consequences, such as increased burden of non-c higher healthcare costs, decreased life expectancy, adverse effects on labor participation, economic productivity and global security implications.^96^ The long-term effects of COVID-19 infection necessitate specialized long COVID care for individuals with cardiometabolic disease.^96^ Early detection through the use of biomarker assays may expedite the implementation of successful preemptive management modalities to effectively reduce the risk of such sequelae.^97,98^ A study conducted proteomic plasma analysis in 92 protein biomarkers collected from 343 hospitalized COVID-19 patients, for the prediction of severe outcomes.^97^ Of note, only 7 were significantly predictive of admission to the intensive care unit (ICU)/death within 28 days of presentation. Of these, ADAMTS13 and VEGFD were significantly predictive of a reduced risk of ICU/death, whereas ACE2, IL-1RA, IL-6, KIM1, and CTSL1 were significantly predictive of an increased risk.^97^ Schroeder and colleagues highlights the significance of proteomic profiling in supporting the initial patient evaluation and their probability of a severe course of COVID-19 disease, and potentially enabling timely management.^97^ Patients with severe COVID-19 also demonstrated increased concentrations of cardiac injury biomarkers, such as N-terminal pro-brain natriuretic peptide (NT-pro-BNP), creatine kinase-MB (CK-MB), and troponins.^99^ Elevated levels of these cardiac markers correlate with increased mortality rates, and should be clinically considered in the management of COVID-19 consequences.^99^ In view of the colossal impact that COVID-19 had on the global population, the relationship between cardiometabolic outcomes and COVID-19 vaccination status were also briefly examined into the potential benefits of vaccination in mitigating long-term cardiometabolic risks associated with COVID-19. We specifically focused on four major cardiometabolic diseases namely, obesity, diabetes mellitus, hypertension and CVD, to provide a deeper understanding of the complex interplay between COVID-19 and cardiometabolic health.

The relationship between the vaccination status and cardiometabolic outcomes has also attracted public attention for its potential efficacy against long COVID.^100^ Of note, a reduced incidence of long COVID was observed among healthcare workers who received two or three doses of the Pfizer-BioNTech mRNA vaccination prior to SARS-CoV-2 infection, compared to those with no vaccination.^101^ The study population was inclusive of 2560 participants, of which 739 (29%) had COVID-19 and 229 (31%; 95% CI, 27.7%-34.5%) long COVID. The prevalence of long COVID varied across the pandemic waves, from 48.1% (95% CI, 39.9%-56.2%) in wave 1 to 35.9% (95% CI, 30.5%-41.6%) in wave 2, and 16.5% (95% CI, 12.4%-21.4%) in wave 3.^101^ There was an association (p<0.001) between the number of vaccine doses and the lower rates of long COVID, a prevalence of 41.8% (95% CI, 37.0%-46.7%) was observed in unvaccinated patients, which reduced to 30% (95% CI, 6.7%-65.2%) with 1 dose, 17.4% (95% CI, 7.8%-31.4%) with 2 doses, and 16.0% (95% CI, 11.8%-21.0%) with 3 doses.^101^ In another study, vaccination only offered partial protection against the long-term effects of COVID-19,^14^ whereas findings from a longitudinal study demonstrated correlation between vaccination and the alleviation of symptoms.^102^ Due to the limited sample size and lack of generalizability, the correlation between COVID-19 vaccination and long COVID remains inconclusive and debatable.^102^

Reports also suggest an elevated risk of myocarditis and pericarditis following the administration of mRNA COVID-19 vaccines, particularly among young males under the age of 25 years.^103^ Notably, the risk was found to be higher after the second dose in comparison to the first or booster doses.^103^ On the contrary, the COVID-19 vaccination was reported to mitigate the likelihood of cardiovascular complications following COVID-19 infection.^104^ In a Norwegian study inclusive of 2,364,651 vaccinated and 1,532,935 unvaccinated individuals, only 1576 (0.09%) of the vaccinated individuals developed at least one cardiometabolic symtom between 90 and 365 days after the date of a COVID-19 positive diagnosis.^104^ There was no record of these symptoms 180 days before SARS-Cov-2 infection, and were therefore identified as long COVID cases.^104^ Notably, lower incidences of arterial and venous thrombotic events were observed in the vaccinated group compared to their unvaccinated counterparts.^101^ Despite reports of several cardiometabolic adverse events associated with COVID-19 vaccines, their widespread use continues due to their effectiveness against the virus.^104^

### 4.2. Obesity post COVID

Obesity exacerbates COVID-19 vulnerability due to shared inflammatory and/or metabolic pathways.^105^ Overweight and obese individuals across all age groups demonstrate an elevated risk of experiencing severe COVID-19 complications.^106^ Keller and colleagues reported a worsening of health outcomes among obese COVID-19 cases, which subsequently increased the occurrences of pneumonia and deaths associated with ARDS.^107^ Among 176,137 cases of COVID-19 hospitalization, 9,383 cases (5.3%) were obese.^107^ The data demonstrates obese cases as younger individuals (66.0 years vs 72.0 years, p < 0.001), with worse CVD risk factor profile (Charlson Index: 4.44 ± 3.01 vs 4.08 ± 2.92, p < 0.001). Based on these findings, obesity must be considered in COVID-19 prevention and risk strategies.^107^ In another study, approximately 20% of all COVID-19 hospitalized patients were obese, 60% presented with obesity in combination with other metabolic comorbidities, such as type 2 diabetes and hypertension.^107^ The rise in the global prevalence of obesity during the pandemic may be due to reduced physical activity and poor dietary patterns.^108^ Findings from a multicenter case-control Spanish study, involving 88 patients with obesity and 176 without, revealed a significant association between obesity and a greater number of long COVID symptoms 2 months after hospital discharge (IRR 1.56, 95% CI 1.24-1.95, p<0.001).^109^ Beydoun and colleagues evaluated 1372 United States participants and highlighted a rise in body mass index (BMI) (Beta (β)= 1.39, 95% CI: 0.74, 2.03) during the pandemic.^110^ The likelihood of having at least one cardiometabolic risk factor and/or chronic illness escalated from pre and post the onset of COVID-19 (OR 1.16, 95% CI: 1.00, 1.36).^110^ In a PREDIMED Plus longitudinal trial, examining the association between adiposity, metabolic syndrome, and the risk of having COVID-19, a higher cardiovascular risk was evident in older people.^111^ The study analysed 6874 participants aged 55 to 75 years, who met the inclusion criteria of being overweight/obese and presenting with metabolic syndrome. The data demonstrates an association between baseline values of body weight, BMI, waist circumference, and waist to height ratio (WHtR) with the risk of having COVID-19.^111^ An association was also evident between the longitudinal increase in body weight (HRadj= 1.01, 95% CI: 1.00-1.03) and BMI (HRadj = 1.04, 95% CI: 1.003-1.08) and an increased risk of having COVID-19. Overall, these findings suggest a protective effect of weight loss against COVID-19.^111^

### 4.3. Diabetes mellitus

Diabetic ketoacidosis (DKA) and hyperosmolarity are major complications observed in patients following COVID-19.^112,113^ Findings from 2 COVID-19 patient cases, aged 30 and 60 years respectively, demonstrated the onset of DKA in the 30 year old, undiagnosed diabetes and no other comorbidities.^112^ However, the second patient aged 60 years who presented with hypertension with no prior history of diabetes subsequently developed both DKA and cerebrovascular accident (CVA) in the hospital,^112^ indicative that DKA is a consequence of COVID-19. Possible disease mechanisms could be due to direct pancreatic injury as a result of SARS-CoV-2 infection and the systemic hyperinflammatory response.^74,114^ Findings from a meta-analysis investigation inclusive of 7 observational studies provides a pooled estimate of the risk of developing incident diabetes following hospital discharge or at least 28 days after the COVID-19 diagnosis compared to matched controls or severity matched influenza/non-COVID-19 acute upper respiratory tract infections (AURI).^115^ The pooled analysis of 5,787,027 subjects from four observational studies,^67,116–118^ showed a 59% higher risk of developing incident diabetes in post-acute COVID-19 phase versus healthy controls (HR=1.59; 95% CI:1.40–1.81, p < 0.001). The remaining three studies^22,89,119^ reported the likelihood of incident diabetes in COVID-19 versus severity matched influenza, pooling data from both mild (n=308,613) and moderate-severe/hospitalized (n=24,090) COVID-19 patients. The high degree of heterogeneity in the pooled estimates may be attributed to variations in demographic characteristics, hospitalization rates and/or disease severity between study subjects.^115^

Rezel-Potts and colleagues analysed electronic records for 428,650 COVID-19 patients, and reported that the net incidence of diabetes mellitus increased in the first 4 weeks after COVID-19 (adjusted rate ratio, RR 1.81, 95% CI 1.51 to 2.19) and remained elevated from 5 to 12 weeks (RR 1.27, 95% CI 1.11 to 1.46) but not from 13 to 52 weeks overall (RR 1.07, 95% CI 0.99 to 1.16).^88^ Acute COVID-19 was associated with a net increased cardiovascular incidence (RR 5.82, 95% CI 4.82 to 7.03) including pulmonary embolism (RR 11.51, 95% CI 7.07 to 18.73), atrial arrythmias (RR 6.44, 95% CI 4.17 to 9.96), and venous thromboses (RR 5.43, 95% CI 3.27 to 9.01). The incidence of cardiovascular disease declined from 5 to 12 weeks (RR 1.49, 95% 1.28 to 1.73).^88^ Findings from a smaller Chinese cohort study which followed 534 COVID-19 hospitalized patients for a median time of 460 days and compared outcomes between severe COVID-19 cases (n=114) and non-severe cases (n=420), revealed sleep disturbance and fatigue as the most common persistent symptoms reported by approximately 18% in both groups.^120^ Additionally, at the 15 month follow up, 3.56% of all participants without previous history of diabetes reported a new fasting blood glucose above 7 mmol/L (126 mg/dL) or HbA1c levels greater than or equal to 6.5%.^120^ In another study, a substantial rise (170%) was observed in the prevalence of hypertension, dyslipidemia, obesity, and prediabetes among T2DM (n=3554) individuals during the pre-pandemic phase compared to the pandemic (n=7430) phase.^94^ The COVID-19 pandemic was effectively investigated to determine the effect of T2DM in Italy using a longitudinal cohort analysis.^121^ Findings from an Italian database of over 200,000 adults participating in a longitudinal cohort study, revealed an increase in the onset of T2DM onset during the pandemic phase (2020-2022) compared to the pre-pandemic phase (2017-2019). The elevation in rate of onset ranges between 4.85 (95% CI, 4.68-5.02) to 12.21 (95% CI, 11.94-12.48) per 1000 person-years, indicative of a rise of 2.5 times.^121^ Furthermore, the doubling time of new T2DM cases shows a substantial reduction, with a shortening time period of 97.12 months (95% CI, 40.51-15375) to 23.13 months (95% CI, 16.02-41.59), indicating a strong link between COVID-19 and T2DM onset.^121^

The findings from a study conducted in the United Kingdom (n=201, mean age 44 years) that included detailed assessments using MRI, showed that at a median follow-up of 140 days following a COVID-19 infection, 98% presented with fatigue, 87% had muscle ache and 88% shortness of breath.^66^ Additionally, evidence of mild organ impairment in the heart (32%), lungs (33%), kidneys (12%), liver (10%) and pancreas (17%) and multi-organ impairment was observed in 25% of the study poipulation.^66^ Therefore, even in young, low risk populations, nearly two-thirds of people have persistent damage of one or more organs 4 months after initial symptoms of SARS-CoV-2 infection, which will have implications for the long-term health of these patients.^66^ Futhermore, the risk of overall diabetes was evaluated in 11 cohorts comprising 47.1 million participants (4.5 million patients with COVID-19 and 42.6 million controls). The random effects model meta-analysis higlights a 64% greater risk of diabetes (RR=1.64, 95% CI: 1.51 to 1.79) with COVID-19.^122^ Data from an Italian case-control study demonstrated a significant increase in the prevalence of gestational diabetes mellitus (GDM) in 2020 compared to 2019 (13.5%: 86/637 versus 9%: 48/533, p=0.01).^123^ The lockdown appeared to be a significant contributor especially during the first trimester, which subsequently increased the incidence of GDM by 34% per month.^123^ It is possible that the stress linked to a positive COVID test, combined with the lack of exercise, and unhealthy behaviors significantly increased the risk of GDM development, thus warranting their consideration by healthcare practitioners when managing pregnant women, especially during future outbreaks.^123^

### 4.4. Hypertension

Angeli and colleagues reported that SARS-CoV-2 infection was associated with 65% increased risk of new onset hypertension (p<0.0001) compared to a control group that did not have the SARS-CoV-2 infection.^124^ Notably, the incidence of new onset hypertension was 9% in the COVID-19 cohort and 5% in the control group.^124^ This may be attributed to the interaction between SARS-CoV-2 spike proteins and angiotensin converting enzyme 2 (ACE_2_) receptors, which may contribute to increased blood pressure both during the acute phase of infection and during recovery.^124^ This interaction indicates that new onset hypertension is a prevalent cardiovascular sequela of COVID-19.^124^ Similar findings were reported by a spanish study, who stated that out of 543 patients who were hospitalized or discharged from an emergency department for COVID-19, 12 (2.2%) patients developed onset hypertension in the following year.^125^ However, due to the lack of a control group, it was unclear whether the incidence exceeds the expected levels.^125^ Likewise, 1.3% of Chinese patients (n=538) developed hypertension approximately 3 months after hospital discharge for COVID-19.^126^ This study included a control group analyses, however it failed to reach statistical significance (p=0.2), and none in the control group who were free of COVID-19 (n=184) developed hypertension in the specified study time frame.^126^ Noteworty, none of these studies formally evaluated new onset hypertension through repetitive blood pressure measurements, but instead relied primarily on medical records and patient reports.^127^ Regardless of the various studies that were conducted on the long-term effects of COVID-19 on pre-existing NCD patients,^41,42,44,52^ only a few collected epidemiological data on the incidence of NCDs post the COVID-19 pandemic.^37,66,128^ Beyond this direct consequence, changes in lifestyle in response to lockdown, social distancing, and the mental stress associated with the pandemic worsened control of multiple risk factors among those with CMD, and those at risk for CMDs.^129^ This increased population-level risk may be attributed to the harsh changes in physical activity, dietary patterns, alcohol intake, and smoking.^129^

## 5. Cardiovascular outcomes of long COVID

Cardiovascular complications such as arrhythmias, ischemic or thrombotic events, inflammation, cardiac arrest, and sudden death are clinical manifestations of long COVID in affected patients.^130^ Pathophysiological conditions that manifested as a consequence of COVID-19 include postural orthostatic tachycardia syndrome (PoTS), angina pectoris and dyspnea on exertion.^131^ The prevalence of PoTS in patients remains uncertain however it is characterized by lightheadedness, palpitations, tremors, generalized weakness, blurred vision, exercise intolerance, and fatigue.^131^ The condition is distinguished by a significant increase in heart rate (exceeding 30 beats per minute) when moving from a supine or seated position to a standing position, in the absence of orthostatic hypotension and frequently misdiagnosed as anxiety, panic disorder, or chronic fatigue syndrome due to shared symptoms.^131^ Since the recent emergence of COVID-19, most studies on long COVID are retrospective, involving limited participants or specific cohorts, lack consistency regarding the timing after initial infection, definitions of dysautonomia or PoTS, and testing protocols.^132^ Furthermore, only 1% of patients present with orthostatic hypotension, whilst others experience reproducible postural symptoms despite normal stand or tilt table test results.^133^ Regrettably, 1 to 10 % of individuals who had COVID-19 may develop cardiac complications such as myocarditis, pericarditis, and arterial blood clot formation, with the initial identification remaining a challenge in management of such cases.^133^ Another commonly experienced symptom is angina pectoris which prompts medical attention.^134^ Medical professionals are required to be vigilant when assessing patients with chest pain, since the SARS-CoV-2 virus was able to induce multiorgan and multisystemic injury, inclusive of the cardiorespiratory, lymphatic and peripheral nervous systems.^134^ A cohort study conducted in Wuhan, China on hospitalized patients with COVID-19 (n=416), with a median age of 64 years (range, 21-95 years), reported that 13.4% (n=14) experienced chest pain.^135^ Findings from an Australian study with a total of 4159 participants, demonstrated that 2,257 patients diagnosed with COVID-19 experienced various symptoms, with 5% reporting chest pain as well.^136^ The etiology of chest pain induced by SARS-CoV-2 infection remains unclear, however, chest pain may possibly be a result of cardiac damage or pleural inflammatory infection.^136–138^ Long COVID dyspnea is a prevalent symptom that considerably impairs an individual’s quality of life.^139^ The precise etiology of long COVID dyspnea remains unclear, with prior research yielding inconsistent findings.^140^ Studies have however failed to identify significant differences between healthy individuals and those experiencing dyspnea following COVID-19 infection.^141,142^ These studies have indicated that dyspnea may have signifcant ramifcations on overall quality of life but is unlikely attributed to a pulmonary limitation to exercise or confounding neurologic, musculoskeletal or fatigue symptoms.^141,142^ Moreover, some individuals reported exercise intolerance, accompanied by indications of irregularities in their circulatory and respiratory systems.^143–145^ Predicting the severity of long-term respiratory symptoms after recovery from the acute phase of COVID-19 remains a challenge.^143–145^ Findings from a prospective study on adult patients admitted in two academic hospitals in Vancouver, Canada with PCR-confirmed SARS-CoV-2 (n=76) during the first wave of COVID-19 (March-June 2020), revealed that 49% and 46% experienced clinically significant dyspnea (baseline score >10 points) at 3 and 12 months long COVID respectively.^139^ Notably, amongst the initial 49% who experienced dyspnea between the 3 and 12 months post COVID-19, 24% experienced a clinical worsening in their dyspnea, 49% remained stable, and 28% demonstrated an improvement.^139^ Patients who exhibited clinically significant dyspnea at the 12-month mark following COVID-19 infection experienced a decline in the quality of their sleep, mood, and overall quality of life, as well as an increase in frailty. These observations were made in comparison to patients who did not experience dyspnea.^139^

## 6. Cardiometabolic patient burdens post the COVID-19 pandemic

The far-reaching psychosocial, socioeconomic and healthcare effects of the pandemic extend beyond physical health, impacting mental well-being, social relationships, and economic stability worldwide.^146^ The WHO recommended collective lockdowns which was essential for slowing the community spread, however, the socioeconomic implications have been substantial, with widespread job losses and temporary suspension of social welfare services.^147,148^ Consequently, the risk of poverty has increased, with a growing number of individuals falling below the at-risk-of-poverty threshold.^148^

### 6.1. Psychological factors

Psychological effects including depression, anxiety, stress, and adjustment disorders have been closely associated with COVID-19, poorer sleep quality, increased substance use, and the consequent increase in antidepressants and opioid use.^149,150^ The prevalence of anxiety, depression, and post-traumatic stress disorder (PTSD) is referred to as “biopsycho-social” phenomena, which encompasses physiological, psychological, and social dimensions of a patient’s life, is significantly higher amongst those with long COVID versus the general population.^151^ Long COVID patients experience viral induced inflammation and immune activation, which can contribute to depression and suicidal behaviours that may be attributed by physiological reasons.^151^ Long COVID can significantly impact the mental health status of individuals who are unable to fulfil their work responsibilities, care for their families, or engage in activities that were previously meaningful to them.^151^ The physical pain and disability associated with long COVID also negatively affects a patient’s mental well-being, with many reporting the onset of insomnia, often due to severe pain.^151^ A good 7-8 hours sleep is crucial for optimising mental health, and thus insomnia has been associated with an increased risk of suicidal thoughts and behaviors.^151^ A comprehensive meta-analysis encompassing 39 distinct studies and involving over 10,000 individuals, reported that approximately 19% with long COVID experienced anxiety as one of their major symptoms, while 8% reported depression.^152^ In England, the onset and trajectory of psychological symptoms in individuals with long COVID and acute COVID-19 infection was investigated, showing an immediate increase in depressive (35%) and anxiety (8.9%) symptoms in both groups following SARS-CoV-2 infection.^153^

**Figure 2.**
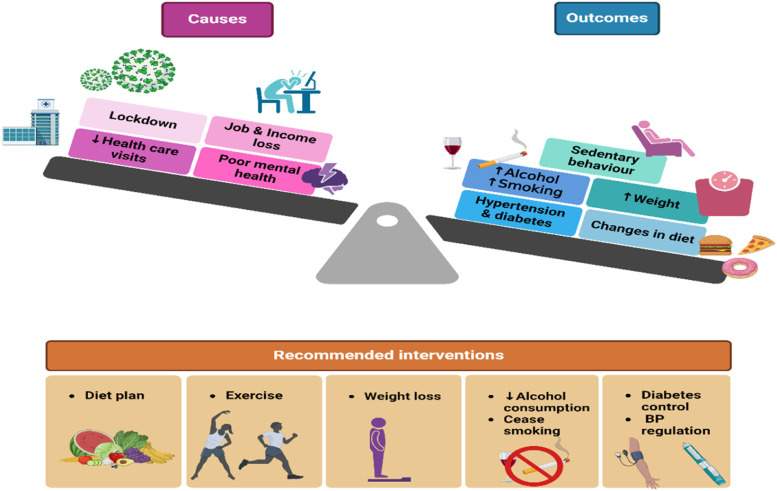
The effects of COVID-19 on cardiometabolic health (Adapted from Kolkailah et al., 2022).^129^ Principal drivers include lifestyle disruptions, psychological distress, and compromised healthcare access, leading to increased blood pressure, deteriorating diabetes control, and heightened cardiovascular risk. However, strategic interventions can offset these detrimental effects. The pandemic’s cardiometabolic impact can be mitigated by leveraging digital health solutions, encouraging healthy behaviors, and addressing socioeconomic disparities. This figure was generated using BioRender (https://biorender.com/).

Several studies investigated the psychological and social effects of COVID-19 on the general population, sourcing data from non-representative samples originating from high-income countries (HICs), low-middle income countries (LMICs), or a combination of both and employing standardized measurement tools.^154–157^ These studies reported varying degrees of prevalence rates of depression, anxiety, and stress.^154–157^ The investigations regarding sociodemographic variables and COVID-19 revealed a correlation between depression and younger age, female gender, elevated levels of exposure, and stigmatization associated with COVID-19.^154^ Similar associations between stress and female gender, single marital status, lack of formal education, religious affiliation, exposure to confirmed or suspected COVID-19 cases, and mandatory quarantine were reported.^158^ In South Africa, COVID-19 infections manifested in mental health issues such as post-traumatic stress disorder, mood disorders, anxiety disorders, phobias, and obsessive-compulsive disorders due to public biological, psychological, and social predispositions.^159^ Noteworthy, findings from the Human Sciences Research Council, confirmed that a significant portion of the South African population experienced adverse psychological effects during the initial lockdown period, with 33% reporting depressive symptoms, 45% experienced fear, and 29% felt a sense of loneliness.^160^ Additionally, many South Africans experienced substantial hardship with many being unreported and undetected during the national lockdown.^161^ Socioeconomic challenges such as unemployment, poverty, limited access to clean water and sanitation, as well as inadequate housing, exacerbated the situation.^162–164^ The primary factors responsible for the negative impact of the COVID-19 pandemic on cardiometabolic health, as well as potential strategies to mitigate these effects is shown in Figure 2. Noteworthy, majority of the existing literature regarding the psychosocial impacts of COVID-19 originates from studies conducted in China, Europe, and the USA, with limited research focusing on other regions, particularly LMICs, despite the severe impact of the pandemic in these areas.^165^ The evaluation of the psychological impacts of the pandemic in these regions faces challenges due to the absence of standardized measures in the local languages which impedes the comparison of results between countries.^165^

### 6.2. Socioeconomic impact

Social, educational, economic, and religious consequences of the COVID-19 pandemic may vary across different countries,^166^ however, it is highly probable that the pandemic exacerbated the rise in poverty and inequality, critically reducing the attainment of sustainable development goals.^166^ Developing and customizing government and partner responses is necessary for effective recovery as well as inclusivity in the process.^167^ Findings from a Birmingham study conducted among 2873 patients (59% female of which 45% were African), revealed that CMD, insurance status, male sex, and higher glucose values were associated with increased odds of all cardiometabolic outcomes.^168^ In contrast, Zhou and colleagues identified socioeconomic status as a significant predictor for CMDs (p <0.001); socioeconomic status was not significantly associated with lifestyle, (p = 0.371), however, lifestyle was a significant predictor for CMD (p = 0.030), thus warranting the importance of conducting a mediation analysis.^169^ Despite the rapid progression of research on the health effects of COVID-19 in low-income countries, there remains a paucity of data regarding the pandemic’s socioeconomic effects.^170,171^ Regardless of comprehensive research studies indicating the influence of unfavourable social health determinants such as unemployment/low income, limited education and racial/ethnic minorities in elevating the risk of acute SARS-CoV-2 infection, there is limited data concerning the association between social determinants and long COVID.^172^ Data from a cohort study confirmed a significant association between Hispanic ethnicity, lower socioeconomic status, and financial insecurity with the onset of long COVID symptoms (p<0.10).^172^ These findings indicate that individuals belonging to these specific demographic groups are more likely to experience persistent symptoms following COVID-19 infection. The data highlighted the need for targeted interventions and support systems to address the health disparities and vulnerabilities faced by these populations in the context of the ongoing pandemic.^172^ Furthermore, Case and colleagues revealed a correlation between financial concerns and diminished health utility and quality of life among individuals recovering from COVID-19.^173^ Notably, the female gender was more susceptible to long COVID symptoms after adjustment, with the exception of persistent symptoms in sensitivity analysis.^174,175^ In contrast, a lower likelihood of experiencing long COVID was shown among individuals with graduate-level education and those residing in urban areas.^176^ An Italian study (n=74294) reported that between 2019 to 2022, obesity-related ORs for diabetes increased from 2.45 (95% CI 1.73-3.47) in 2019 to 3.02 (95% CI 2.09-4.35) in 2022, while hypertension increased from 2.86 (95% CI 2.28-3.58) to 3.64 (95% CI 2.87-4.61).^177^ The incidence of hypertension in 2022 was associated with lower education levels, with only middle (OR = 1.09, p = 0.010) or high school diplomas (OR=1.12, p = 0.001), demonstrating significantly higher ORs than individuals with higher education versus non-significance when compared to the 2019 data.^177^ Conversely, the incidence of diabetes associated with lower education levels remained stable and significant in both years.^177^ Findings from a systematic review indicate that during the COVID-19 lockdown, fasting blood glucose was significantly higher in the upper middle-income (mean difference: 5.10; 95% CI: 2.92, 7.27), and high-income groups (mean difference: 6.03; 95% CI: 0.04, 12.02) compared to lower income groups.^178^ This is indicative that prolonged COVID-19 lockdown is associated with worsening anthropometric and glycemic results in an individual with type 2 diabetes.^178^ Of note, there were no significant changes to weight, waist circumference, or HbA1C over the lockdown period.^178^ High SES is interrelated to poorer anthropometric and glycemic outcomes after prolonged confinement, with adult type 2 diabetics having received less effective care during the lockdown, especially in HICs.^178^ The disparate impact of the pandemic on socioeconomically disadvantaged communities may be attributed to higher concentrations of minority ethnic groups, increased prevalence of chronic health conditions, occupational exposure risks, reliance on public transportation, crowded or multigenerational living arrangements, and limited access to healthcare services.^179^ It is imperative that forthcoming healthcare policy recommendations encompass the multifaceted aspects of inequality, including gender, socioeconomic status, and professional background, when addressing the treatment and management of long COVID.^179^

## 7. Management of cardiometabolic outcomes

Clinically there remains no universally accepted treatment for long COVID.^180^ Therapeutic approaches continue to be tailored to manage individual patients, with the primary objective of managing disease progression and preventing hospitalization.^181^ Identifying and understanding the potential biological pathways that could be targeted with repurposed medications and innovative therapeutics to address long COVID phenotypes is necessary.^180^ Despite the investigations surrounding the use of metformin, ivermectin, and fluvoxamine as potential therapeutics to mitigate or prevent severe COVID-19 related complications, a double-blind, randomized controlled trial reported that none demonstrated significant efficacy in reducing hypoxemia, emergency department visits, hospitalizations, or mortality associated with COVID-19.^180^ Cardiac rehabilitation programs serve as valuable supplementary measures in preventive medicine to mitigate the risk of COVID-19-induced cardiac complications.^181^ There is however a lack of conclusive evidence supporting a specific treatment for long COVID associated cardiac damage.^181^ Rehabilitation might not be suitable for individuals who have experienced severe COVID-19 and have significant pulmonary or cardiac impairments. It is therefore important to recognise the potential overlap between cardiopulmonary symptoms of premature atrial contractions and deconditioning due to acute COVID-19 infection.^182^ Graduated exercise regimens, including recumbent or semi-recumbent exercises such as swimming, are recommended for individuals with significant postural symptoms.^182,183^ Its possible that the national lockdown and consequent home confinement associated with the COVID-19 pandemic exacerbated unhealthy lifestyle choices such as poor diets and physical inactivity.^184^ Physical activity and exercise are instrumental in the prevention and control of NCDs such as diabetes and hypertension.^178^ Moreover, pharmacological and non pharmacological interventions such as compression stockings, midodrine and beta-blockers may assist patients experiencing autonomic dysregulation.^182,183^ Unfortunately, the most disadvantaged individuals, who often have higher rates of comorbidities, limited access to healthcare, and challenges in returning to full-time employment, are more likely to experience the most severe consequences of long COVID.^185^ Low- and middle-income countries have lower coverage of adequate social protection measures, such as social support grants or social relief of distress grants, which could help mitigate the ongoing negative impacts of the pandemic.^185^ There is greater access to COVID-19 vaccines in HICs that have received proportionately more vaccine doses, enabling a wider community vaccination.^185^ Data emanating from a 2022 scoping review showed that out of 59 registered clinical trials focussing on long COVID therapeutics, only 15 included patients from low-to-middle-income countries (LMICs).^186^ This limited representation hinders the ability to comprehensively assess the efficacy and applicability of potential treatments across diverse settings and populations.^186^ It is thus crucial to address this disparity, as LMICs currently lack access to innovative COVID-19 therapeutics and will face challenges in obtaining long COVID treatments.^186^

## 8. Conclusion

Our findings confirm a significant number of individuals suffering from CMD developing from damage during acute infection, as well as other manifestations of long COVID both nationally and internationationally. Long COVID outcomes, including diabetes and hypertension, have been observed in low-risk patients who experienced mild symptoms, underscoring the necessity for vigilance even in young, healthy populations. Although studies have indicated improvement over time, continued monitoring of long COVID patients will be essential to better characterize long-term outcomes. This review contributes to the growing body of literature summarizing post-acute cardiometabolic outcomes of COVID-19, with a focus on outcomes occurring more than 3 month after acute illness. While current evidence remains limited in quantity and quality, this analysis will encourage clinicians to be cognizant of potential risk factors and can serve as a starting point for future investigations. Given the compelling evidence suggesting a causal relationship between SARS-CoV-2 infection and the development of persistent symptoms and chronic multisystem syndrome (CMS), it is imperative that we maintain robust public health interventions to mitigate viral transmission. Proactive preparation of the healthcare systems to manage the anticipated surge in CMS-associated diseases in the foreseeable future is crucial due to the the burgeoning number of individuals affected by long COVID. Notably, in Africa, the healthcare system has been disrupted, causing delayed diagnosis and treatment of long COVID as well as inadequate infrastructure and limited healthcare workers. Particularly, in South Africa there is a need for integrated chronic care models that can control blood pressure and glucose management in patients with cardiometabolic multimorbidity. This looming public health crisis necessitates a substantial expansion of both healthcare services and social support systems, considering the significant medical attention required and the potential reduction in work capacity experienced by affected individuals. Furthermore, there is an urgent need for intensified research efforts to elucidate the precise mechanisms underlying cardiometabolic progression in COVID-19 patients, both during active infection and post-viral clearance. This entails a deeper understanding of the role of immune and inflammatory factors in these processes. Identifying and therapeutically targeting the pathological pathways driving this biological cascade will be paramount in developing effective treatment strategies for long COVID patients.

## Data Availability

Data sharing not applicable to this article as no datasets were generated or analysed during the current study.*
